# Diagnostic, predictive, and therapeutic approaches for impacted canines: a systematic review and meta-analysis

**DOI:** 10.1186/s12903-026-08072-5

**Published:** 2026-03-10

**Authors:** Wang Yi, Johari Yap Abdullah, Umi Mardhiyyah Mat Ali, A S M Rafiul Haque

**Affiliations:** 1 School of Dental Sciences, Universiti Sains Malaysia, Kampus Kesihatan, Kubang Kerian, 16150 Kelantan Malaysia; 2 Dental Research Unit, Center for Transdisciplinary Research (CFTR), Saveetha Institute of Medical and Technical Sciences, Saveetha Dental College, Saveetha University, Chennai, India; 3 Orthodontic Unit, School of Dental Sciences, Universiti Sains Malaysia, Kampus Kesihatan, Kubang Kerian, 16150 Kelantan Malaysia; 4 Dept. of Dental Anatomy, Udayan Dental College, House-140, Ward-09, Hoseniganj, Ghoramara, Boalia, Rajshahi Bangladesh

**Keywords:** Impacted maxillary canine, Cone-beam computed tomography (CBCT), Artificial intelligence in dentistry, Diagnostic imaging

## Abstract

**Objective:**

Maxillary canine impaction affects approximately 1–3% of the population and presents diagnostic, prognostic, and therapeutic challenges. The clinical utility of artificial intelligence (AI) in this context remains uncertain. We synthesized evidence on diagnostic, predictive, and therapeutic approaches and situated AI-based methods alongside established strategies.

**Methods:**

PRISMA 2020-guided searches of PubMed, the Cochrane Library, Web of Science, and Embase (2015–2024) identified 28 eligible studies, grouped into diagnostic, predictive, and therapeutic domains. Risk of bias was assessed using domain-appropriate instruments. Meta-analyses were conducted by domain (no cross-domain pooled effect); diagnostic accuracy synthesis used hierarchical bivariate/HSROC models to accommodate threshold variability.

**Results:**

Diagnostic performance was moderate-to-substantial. CBCT was associated with higher diagnostic accuracy than two-dimensional panoramic radiography (OR 3.13, 95% CI 2.34–4.94; I² = 0.0%, *p* = 0.121). AI-related diagnostic estimates were reported as higher in a small subset of studies; however, limited sample sizes, heterogeneity, scarce external validation, and inconsistent reporting preclude clinical inference and warrant cautious interpretation. Predictive findings highlighted spatial parameters, particularly inter-tooth/inter-root contact distance. Therapeutic evidence suggested potential benefit of active interceptive interventions over conservative management, but most studies were short-term, heterogeneous, and predominantly non-randomized.

**Conclusions:**

CBCT and reproducible spatial indicators provide a practical framework, with imaging guided by radiation protection principles (ALARA). Current evidence does not support AI-based methods as clinically decisive; at best, they may serve as adjunctive, hypothesis-generating tools. Larger prospective studies with longer follow-up, standardized reporting, and rigorous external validation and calibration are needed.

**Clinical Trial Registration:**

PROSPERO Registration Number: CRD420251239971

**Supplementary Information:**

The online version contains supplementary material available at 10.1186/s12903-026-08072-5.

## Introduction

Although they comprise only 1% to 3% of all tooth impaction cases, impacted canines represent one of the most clinically significant disturbances of dental eruption [1]. Their presence may result in dental crowding, resorption of adjacent roots, and periodontal tissue compromise, which can have lasting effects on oral function and facial aesthetics [2]. In complex presentations, the three-dimensional spatial orientation of the canines is often highly irregular, and their interactions with surrounding anatomical structures vary markedly among individuals. These factors pose substantial challenges for accurate diagnosis and effective treatment planning. Conventional imaging modalities, such as panoramic radiographs, lateral cephalograms, and cone-beam computed tomography (CBCT), provide valuable diagnostic information; however, interpretation remains heavily dependent on clinical expertise. Diagnostic consistency and accuracy can be particularly difficult to achieve in cases involving complex spatial relationships, multiple impactions, or suspected root resorption [3]. Orthodontic traction remains the primary treatment modality; however, there is no standardized, evidence-based framework for determining the optimal direction or magnitude of traction forces. Individual variability further influences treatment duration, patient adherence, and the long-term stability of outcomes [4, 5]. As a result, precise localization, risk stratification, and individualized management of impacted canines remain persistent challenges in clinical practice.

Efforts to improve diagnostic precision and treatment effectiveness have led to investigations into optimized imaging techniques and refinements in traction mechanics. Kumar et al. [6] demonstrated that three-dimensional CBCT offers superior spatial characterization of impacted canines compared with two-dimensional imaging and may enhance diagnostic accuracy. Nonetheless, diagnostic performance continues to be influenced by clinician experience, and agreement among evaluators remains suboptimal in complex cases. In therapeutic management, Cruz et al. [7] proposed improved selection of traction direction and force magnitude, which reduced the incidence of root resorption and improved treatment success. However, in real-world clinical scenarios, particularly those requiring prolonged treatment, patient adherence frequently becomes a limiting factor. Complex impaction cases often involve irregular anatomical constraints, variable patient cooperation, and substantial uncertainty, making it difficult for these technical refinements to meet the demands of precision. A systematic review by Grisar et al. [8] further highlighted long-standing issues in the field, including the lack of standardized diagnostic criteria and insufficient individualization of treatment planning, particularly in complex clinical presentations. Disturbances in maxillary canine eruption occur along a clinical continuum ranging from delayed eruption and ectopic displacement to impaction or clinical absence. Population-based evidence indicates that dental anomalies are common in growing individuals, with maxillary canine displacement and impaction among the most prevalent findings and frequently associated with other anomalies, such as hypodontia and transposition [9]. Clinically, missing or impacted canines may lead to esthetic compromise, occlusal discrepancies, space loss, midline deviation, and an increased risk of root resorption of adjacent incisors, often resulting in prolonged and complex orthodontic management [9, 10]. These findings emphasize the importance of early identification and risk stratification of eruption disturbances to mitigate downstream complications.

Recent advances in geometric morphometrics (GMA) and artificial intelligence (AI) have introduced new opportunities for the diagnosis and management of impacted canines. GMA provides a quantitative framework for analyzing the three-dimensional morphology of dental and skeletal structures and offers enhanced discriminatory capability in morphologically complex cases. Mucedero et al. [11] demonstrated that GMA improves the accuracy of predicting canine position in cases involving multiple impactions or irregular anatomic configurations. However, its broader clinical adoption is limited by the need for specialized technical expertise and the scarcity of large-scale validation studies. At the same time, AI models based on deep learning have developed rapidly within dental imaging. Minhas et al. [12] reported high efficiency in complex imaging assessments. Despite these promising developments, both GMA and AI approaches remain constrained by issues such as limited dataset size, sampling bias, insufficient model interpretability, and a lack of external validation, which restrict their readiness for routine clinical decision-making.

In parallel, healthcare research increasingly adopts predictive and data-driven modeling to support diagnosis, prognosis, and treatment planning in complex biological systems. Recent studies highlight how machine learning and statistical modeling frameworks can integrate heterogeneous clinical variables to enhance decision-making, while also addressing uncertainty and interpretability in medical contexts [13, 14]. Although developed for other disease domains, these approaches reflect a broader methodological shift that is relevant to predictive strategies for orthodontic conditions with multifactorial etiology and variable outcomes. Methodological advances in AI and predictive modeling demonstrate how complex, nonlinear systems can be analyzed effectively even when data are limited or heterogeneous. For example, digital twin-based diagnostic frameworks have been proposed to transfer diagnostic knowledge across domains while preserving interpretability and robustness, highlighting the potential of virtual representations to support complex diagnostic tasks [15]. Similarly, deep learning approaches grounded in physical or mechanistic modeling have shown improved reliability and generalization by integrating domain knowledge with data-driven learning, particularly in systems characterized by nonlinear behavior [16, 17]. In addition, uncertainty-aware and imprecision-tolerant learning strategies have been developed to handle ambiguous or overlapping patterns better, reducing the risk of overconfident predictions in complex datasets [18]. Optimization-driven machine learning frameworks have demonstrated efficiency in navigating large parameter spaces and improving predictive performance under constrained experimental conditions [19], while reliability-focused modeling has further emphasized the importance of robustness and failure-sensitive prediction in complex systems [20]. Although some studies originate outside dentistry, they still provide conceptual insight rather than direct clinical evidence and illustrate computational principles that may be adapted to support diagnostic and predictive tasks in orthodontics, including the assessment of impacted canines.

Although a substantial body of research has addressed diagnostic methods, risk prediction, and therapeutic strategies for impacted canines, there remains a lack of systematic evidence comparing the relative performance of conventional methods and emerging technologies across different stages of clinical management. In diagnostic assessment, it remains unclear whether AI models can reliably match or exceed traditional methods across imaging modalities and populations. In risk prediction, the consistency and generalizability of traditional geometric indicators and AI-based models remain uncertain. In therapeutic management, quantitative evidence is limited regarding the real-world benefits of different strategies, including traction success rates, complication rates, and treatment durations. In response to these gaps, the present study conducts a systematic review and meta-analysis of diagnostic, predictive, and treatment-management approaches for impacted canines. This study further compares the performance of AI-based techniques with conventional methods, providing comprehensive evidence supporting precision diagnosis and individualized clinical management.

This review, therefore, adopts a domain-stratified approach, synthesizing conventional diagnostic, predictive, and therapeutic evidence alongside emerging AI-based methods, with the explicit aim of contextual comparison rather than direct equivalence.

## Methods

### Design of the systematic review and meta-analysis

This study was conducted within a structured framework for systematic reviews and meta-analyses and followed the PRISMA 2020 reporting guidelines [21]. The scope and eligibility criteria were defined a priori according to the PICOS framework [22]. The overall design centered on comparing AI methods with conventional approaches in the diagnosis and management of impacted canines. Three categories of studies were prespecified for inclusion: diagnostic investigations, predictive model studies, and therapeutic or management studies. Diagnostic studies were required to evaluate the accuracy of detecting, identifying, or classifying impacted canines. Predictive studies were required to develop or validate models to forecast clinical outcomes, including treatment success, complications, treatment duration, and recurrence. Therapeutic studies were required to compare different treatment or management strategies, including AI-assisted decision-making or parameter selection relative to traditional approaches. The objective of the review was to integrate quantitative and qualitative evidence to assess the relative strengths, evidence gaps, and clinical applicability of different methods in the diagnosis and management of impacted canines, thereby providing evidence-based support for individualized care, particularly in complex cases.

Given the differing levels of clinical maturity between conventional approaches and AI-based methods, the review was designed to analyze these domains separately within a unified framework. The objective was not to directly compare or rank conventional and AI approaches, but to evaluate the current state, methodological robustness, and clinical readiness of each evidence stream using domain-appropriate synthesis and interpretation. The inclusion of AI-based studies was intended to contextualize emerging evidence and identify research gaps, rather than to establish clinical effectiveness or to replace conventional diagnostic approaches.

### Search strategy

A systematic search was conducted in PubMed, the Cochrane Library, Web of Science, and Embase for studies published between 2015 and 2024. Search terms were constructed around key concepts related to impacted canines and the three target study types, while also incorporating methodological dimensions. Core free-text terms and controlled vocabulary included, but were not limited to, impacted canine, diagnosis, accuracy, prediction, prognosis, model, treatment, management, machine learning, deep learning, cephalometric analysis, CBCT, and panoramic radiography (OPG). These terms were supplemented using MeSH and Emtree expansions and stem-based processing. Boolean logic (AND/OR) and proximity operators were applied to achieve both high sensitivity and high precision. Searches were restricted to English-language publications. After deduplication, reference lists of included studies and relevant reviews were screened to ensure comprehensive coverage of diagnostic, predictive, and therapeutic evidence related to impacted canines.

The search period was restricted to studies published between 2015 and 2024 to reflect the era of widespread clinical adoption of cone-beam computed tomography and the emergence of AI-based diagnostic and predictive methods relevant to contemporary orthodontic practice. Studies published in 2025 were not included because the final literature search was completed prior to the comprehensive indexing of 2025 publications across all databases. The complete electronic search strategies, including full Boolean search strings and database-specific syntax for PubMed, Embase, Web of Science, and the Cochrane Library, are provided in Supplementary Table S1. This ensures transparency and reproducibility of the literature search in accordance with PRISMA 2020 recommendations.

### Criteria for considering studies for the review

Studies were eligible if they met the following general criteria: human subjects; full text available in English; sample size of at least 30 participants; extractable or convertible effect estimates with measures of uncertainty such as 95% confidence intervals; and a study design that qualified as a randomized controlled trial, cohort study, or case-control study. Each study also had to clearly fall into one of the three predefined categories: diagnostic, predictive, or therapeutic. Diagnostic studies were required to report sufficient data for constructing a 2 × 2 contingency table or to provide accuracy indicators such as sensitivity, specificity, the area under the curve (AUC), or the diagnostic odds ratio (DOR). Predictive model studies were required to develop or validate an AI-based or traditional statistical model for prespecified clinical outcomes, with reported measures of discrimination (AUC or c-index) and, when available, calibration metrics such as calibration slope, intercept, or Brier score. Studies with external validation were prioritized. Therapeutic studies were required to compare treatment or management strategies, including AI-assisted approaches, and to report effect sizes such as odds ratios (OR), risk ratios (RR), or hazard ratios (HR). Exclusion criteria included non-human studies; research restricted to special populations with specific anomalies such as cleft lip and palate; insufficient sample size or single-case reports lacking essential parameters; and studies that provided only morphological descriptions without evaluating diagnostic, predictive, or therapeutic methods.

For studies employing AI-based methods, additional eligibility criteria were applied. Eligible AI studies were required to report quantitative performance metrics such as sensitivity, specificity, AUC, or convertible odds ratios; clearly describe the training-validation strategy (internal validation, cross-validation, or external validation); and include a dataset of sufficient size to permit stable effect estimation. Studies lacking transparent validation procedures or providing purely descriptive AI outputs were included for qualitative synthesis only.

A minimum sample size of 30 participants was required to reduce the influence of very small case series and exploratory studies, which are more susceptible to unstable estimates and exaggerated effect sizes. This threshold was applied as a pragmatic methodological safeguard to enhance the robustness and comparability of quantitative synthesis across heterogeneous study designs. While this criterion may have excluded some smaller studies, it was intended to balance inclusiveness with statistical reliability.

Systematic reviews identified during screening were excluded as primary studies from the quantitative synthesis. When cited, they were used exclusively for contextual comparison and narrative discussion, and no data from their included primary studies were extracted to avoid duplication or violation of the predefined inclusion period. Reasons for exclusion at the full-text eligibility stage are reported with exact counts in the PRISMA 2020 flow diagram.

#### Eligibility criteria and research questions PICOS framework

The research question and eligibility criteria were defined using the PICOS framework:

Population (P): Patients with impacted permanent maxillary canines, regardless of age or sex.

Intervention (I): Diagnostic, predictive, and management approaches for impacted canines. These included imaging methods such as cone-beam computed tomography and panoramic radiography, clinical or radiographic predictors, artificial intelligence-based models, and therapeutic or interceptive interventions.

Comparator (C): Alternative imaging methods, predictive indicators, or management strategies, including conservative management or standard care, when reported.

Outcomes (O): Diagnostic accuracy outcomes, predictive performance measures, and clinically relevant management outcomes, including eruption and treatment success, as reported in the included studies.

Study design (S): Diagnostic accuracy studies, randomized controlled trials, and prospective or retrospective observational studies that met the predefined eligibility criteria.

### Data extraction

Data extraction was performed independently by two reviewers to ensure methodological rigor and completeness. Extracted information included general study characteristics (authors, year, country or region, study design, sample size, and demographic features) and methodological variables such as the use of AI; imaging modality (CBCT, panoramic radiographs, or cephalometric radiographs); model or algorithm type (such as convolutional neural networks, support vector machines, random forests, or logistic regression); threshold definitions; training-validation strategy; and the presence of external validation. Outcomes were extracted according to study type. For diagnostic studies, data included TP, FP, FN, TN, or convertible accuracy indicators such as sensitivity, specificity, AUC, and DOR. For predictive studies, discrimination metrics (AUC or c-index), calibration measures, external validation status, and, when available, decision curve analysis (DCA) were recorded. For therapeutic studies, extracted data included outcome definitions (such as treatment success, complications, or treatment duration), event counts in comparison groups, and effect estimates (OR, RR, or HR with 95% confidence intervals). When information was incomplete or uncertain, study authors were contacted, or predefined rules were applied, and the impact of these decisions was assessed through sensitivity analyses. Unresolved discrepancies were discussed with a third expert reviewer to reach a consensus. For predictive model studies, discrimination metrics were extracted where available; however, calibration measures such as calibration slope, intercept, or Brier score were inconsistently reported and therefore could not be synthesized quantitatively in most cases.

### Assessment of the methodological quality and risk of bias

Risk of bias was independently assessed by two reviewers using tools appropriate for each study type, with written justification for each judgement. Diagnostic studies were assessed using QUADAS-2, covering patient selection, index test, reference standard, and flow and timing. Therapeutic studies were assessed using RoB 2.0 for randomized controlled trials and ROBINS-I for nonrandomized studies, with attention to domains including confounding, intervention classification, deviations from intended interventions, missing data, outcome measurement, and selective reporting. For predictive model studies, the PROBAST framework was referenced across its four domains: participants, predictors, outcomes, and analysis, to qualitatively evaluate methodological soundness without deviating from the original review protocol. To mitigate limitations inherent in two-reviewer assessments, a methodological consultant also reviewed studies deemed high risk or with critical uncertainties, and final decisions were reached through consensus. In the statistical synthesis, prespecified sensitivity analyses and stratified reporting were conducted to assess the robustness of the conclusions and enhance interpretability.

### Statistical analysis

Meta-analyses were performed using Stata 17.0 and RevMan 5.4, with supplementary calculations conducted in R when necessary. Analyses were first stratified by study type: diagnostic, predictive, and therapeutic. Within each stratum, comparisons between AI-based methods and conventional approaches were treated as predefined subgroups and potential interaction factors. Heterogeneity was quantified using the Q test and the I² and τ² statistics. A random-effects model was applied when I² exceeded 50%; otherwise, fixed-effect estimates were reported for comparison. Sensitivity analyses, including leave-one-out procedures, were conducted to evaluate the robustness of results. For diagnostic studies, pooled sensitivity, specificity, and AUC were estimated using a bivariate random-effects model and the HSROC framework. Threshold effects and DOR were assessed when feasible. For predictive modelling studies, quantitative pooling was performed only when the clinical question, outcome definitions, and discrimination metrics were sufficiently comparable across studies, with AUC and c-index as the primary discrimination measures. To improve interpretability, models with internal validation only were reported separately from externally validated models. Calibration information was extracted whenever reported, including calibration slope, calibration intercept, and Brier score. However, because calibration reporting was generally limited and inconsistent across studies, robust quantitative synthesis was not feasible in most cases. Calibration evidence was therefore summarised qualitatively rather than statistically pooled. For therapeutic studies, pooled OR, RR, or HR with 95% confidence intervals were calculated. Subgroup analyses or meta-regression were performed based on study design, intervention type (AI-assisted versus traditional), and imaging modality to explore potential sources of heterogeneity. Publication bias was assessed using Deeks’ funnel plot for diagnostic studies and Egger’s or Begg’s test for predictive and therapeutic studies. Statistical significance was defined as two-tailed *p* < 0.05, with emphasis placed on effect size, precision, and heterogeneity rather than p values alone.

Given the conceptual and methodological differences among diagnostic accuracy studies, predictive modeling investigations, and therapeutic outcome studies, meta-analyses were conducted primarily within each outcome domain. Odds ratios were used as a common effect measure because they are widely reported across study types and outcomes; however, pooled estimates were interpreted only within their respective clinical domains. An overall pooled odds ratio was calculated solely for exploratory descriptive purposes and is not intended to support unified clinical inference across heterogeneous study designs.

Given the limited number of AI-based studies and substantial heterogeneity in model architectures, feature inputs, and validation strategies, a quantitative synthesis of AI outcomes was undertaken with caution. AI subgroup analyses were treated as exploratory, and greater emphasis was placed on direction and consistency of effects rather than precise pooled estimates. Where methodological heterogeneity precluded robust pooling, findings were synthesized narratively.

For diagnostic accuracy studies, threshold variability across studies was addressed using an HSROC model, which jointly estimates sensitivity and specificity while allowing for study-specific thresholds. This approach obviates the need for explicit threshold standardization and accounts for implicit differences in diagnostic cut-off definitions. Threshold effects were therefore incorporated within the model structure rather than analyzed as a separate covariate.

Statistical significance was interpreted in conjunction with effect size magnitude, confidence intervals, and heterogeneity metrics, rather than p-values alone. Given anticipated heterogeneity across study designs and outcome domains, subgroup and domain-specific analyses were prioritized for clinical interpretation, while overall pooled estimates were treated as descriptive summaries. Studies deemed to have a high risk of bias were not excluded a priori from the meta-analysis to preserve transparency and avoid selective exclusion, particularly given the limited number of eligible studies in some outcome domains. Instead, the potential influence of study quality was evaluated through sensitivity analyses and cautious interpretation of pooled estimates. Risk-of-bias assessments were therefore used to inform interpretation rather than as strict exclusion criteria.

Publication bias was evaluated using visual inspection of funnel plots and quantitative tests, including Egger’s regression test and Begg’s rank correlation test, where a sufficient number of studies (≥ 10) were available. A p-value < 0.05 was considered indicative of potential small-study effects.

In studies reporting multiple relevant comparisons, separate effect estimates were extracted only when they were based on independent participant groups or non-overlapping outcome definitions. Where multiple estimates originated from the same study, labels (e.g., “Author_year_1”, “Author_year_2”) were used solely for graphical clarity in forest plots. No participant-level data were double-counted, and correlated outcomes were not pooled simultaneously. Given the limited number of such cases and the absence of shared samples, a three-level meta-analytic model was not required.

## Results

### Search results

**Fig. 1:**
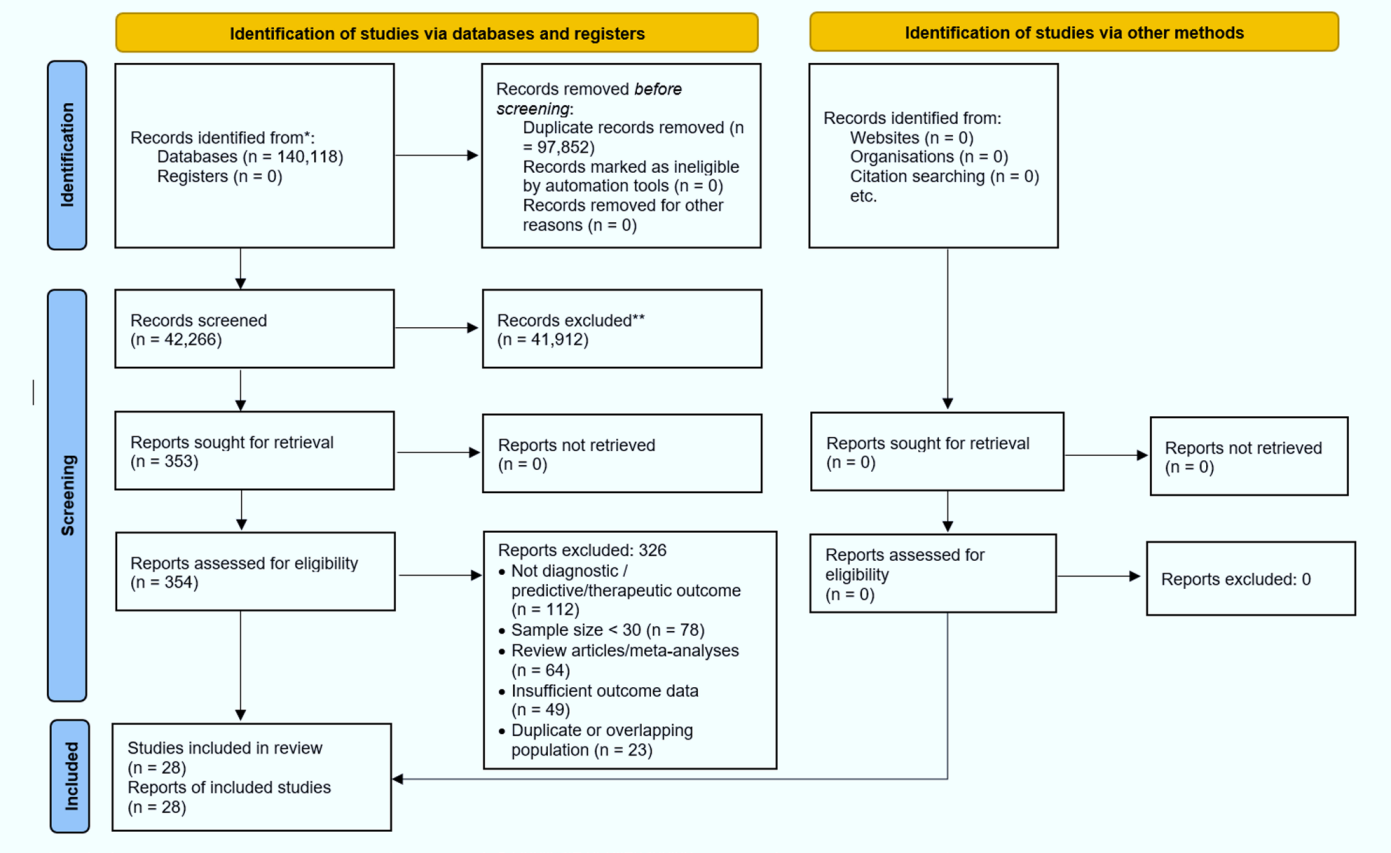
PRISMA flow diagram

Following the predefined eligibility criteria, database searching, deduplication, and multi-stage screening, a total of 28 independent studies [23–50] were included in the final statistical synthesis. The detailed selection process is summarized in Fig. 1.

### Study characteristics

**Fig. 2:**
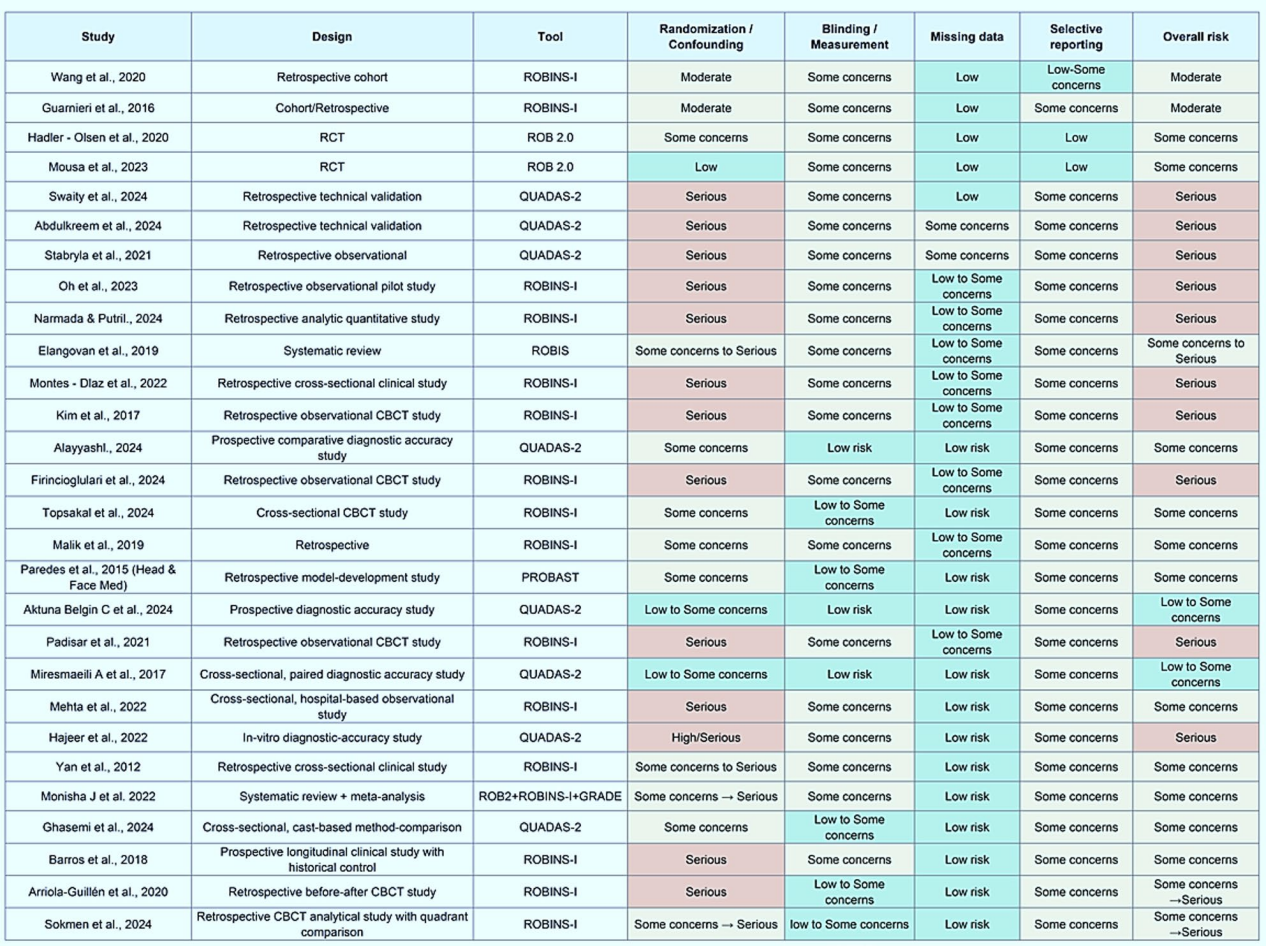
Color-coded diagnostic performance map

The included studies were conducted across multiple countries and research centers and comprised cross-sectional analyses, retrospective and prospective cohort studies, case-control designs, and randomized controlled trials. They covered a range of clinical scenarios involving diagnosis, risk prediction, and treatment or management of impacted canines. Most studies employed CBCT or panoramic radiography as the primary imaging modality; a smaller number incorporated cephalometric radiographs or multimodal imaging. Several studies used deep learning-based AI models for automated detection or classification. Despite heterogeneity in study design, sample sources, imaging modalities, and outcome definitions, all studies could be systematically analyzed within the overarching framework of methodological comparison. For diagnostic studies, CBCT consistently outperformed traditional two-dimensional imaging (pooled OR = 3.13; 95% CI 2.34–4.94; I² = 0.0%; *p* = 0.121), with high inter-study consistency. In the AI-assisted diagnostic subgroup, two studies reported larger pooled effect sizes (pooled OR = 9.43; 95% CI 3.38–26.33; *p* < 0.001), suggesting a potential advantage of AI in identification and classification tasks for impacted canines, although the evidence base was small and confidence intervals were wide. Results are presented in Fig. 2. The detailed characteristics and extracted outcome data of the included studies are provided in Supplementary Table S2, including study design, outcome domain (diagnostic, predictive, or therapeutic), imaging or intervention type, extracted effect estimates, and risk-of-bias assessments.

### Methodological quality and risk of bias

**Fig. 3:**
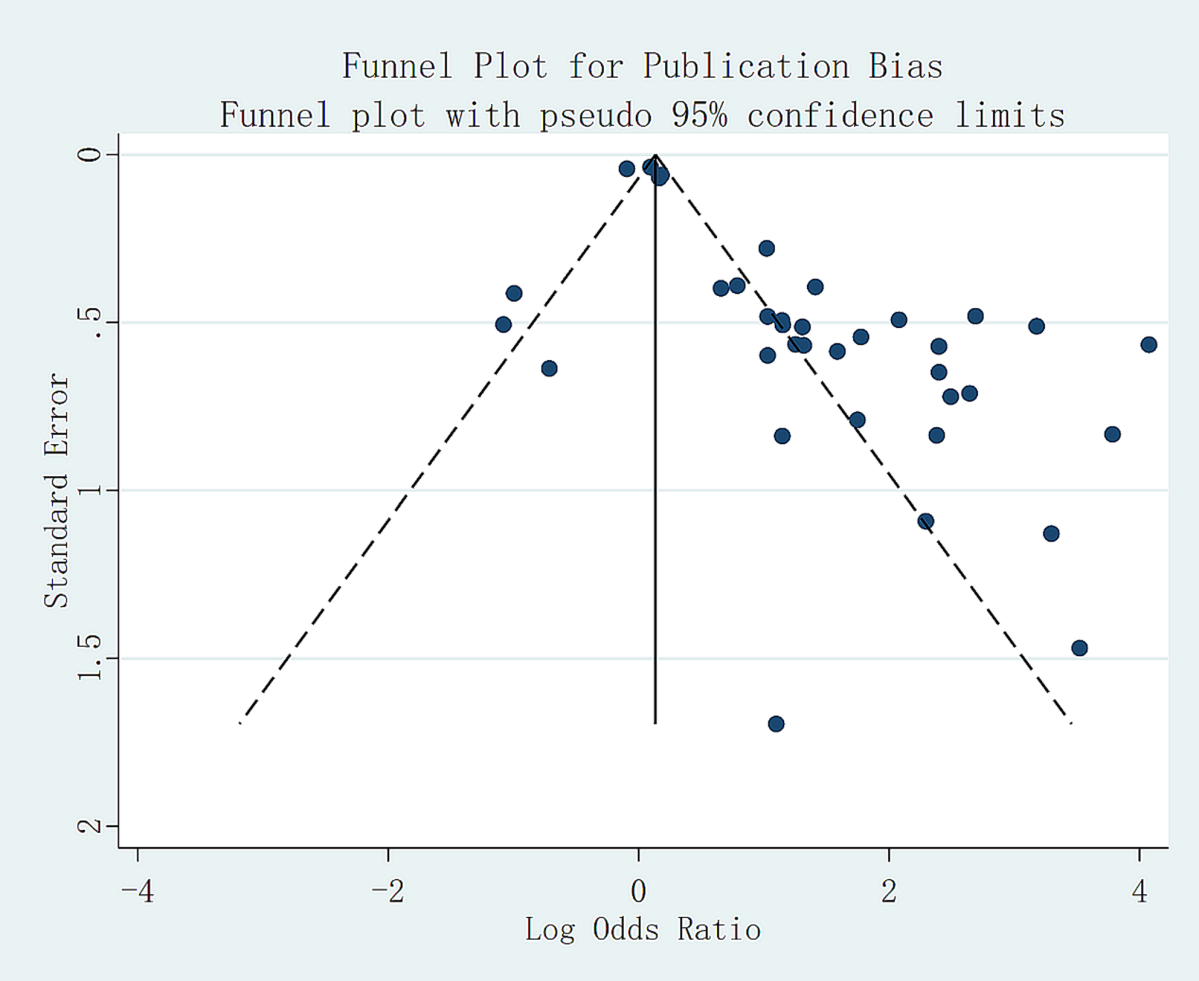
Risk-of-bias summary plot

Risk of bias was assessed using tools appropriate for each study type: QUADAS-2 for diagnostic accuracy research (with a minority evaluated using RoB 2.0), ROBINS-I for predictive model studies, and RoB 2.0 or ROBINS-I for therapeutic studies, depending on design characteristics. Several studies exhibited concerns in key domains, particularly regarding participant selection and analytical methods. Assessment of publication bias showed generally symmetrical funnel plots, with most studies falling within the confidence limits, indicating a low overall likelihood of publication bias. A small number of studies appeared outside the limits, likely attributable to small sample sizes, differing case compositions, or genuine heterogeneity, with minimal impact on the overall direction of the conclusions. Results are summarized in Fig. 3, which presents the risk-of-bias assessment across the included studies and illustrates domain-level judgments regarding participant selection, index test or predictor definition, outcome measurement, and analytical methodology. The figure highlights areas of low, unclear, and high risk of bias across diagnostic, predictive, and therapeutic studies, thereby providing an overview of the internal validity of the evidence base evaluated in this review. Visual inspection of the funnel plot (Fig. 3) suggested asymmetry consistent with potential publication bias, which may partially reflect small-study effects and the predominance of non-randomized designs. Quantitative assessment revealed evidence of small-study effects (Egger’s test *p* < 0.05), whereas Begg’s test showed weaker evidence of asymmetry, suggesting that publication bias cannot be excluded.

### Effect estimates and comparison between AI and traditional methods

#### Diagnostic studies

**Fig. 4:**
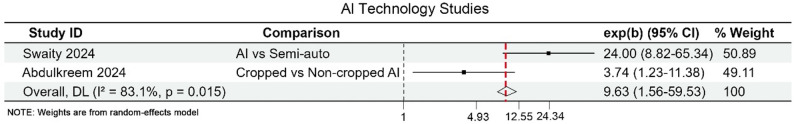
Diagnostic effect comparison (AI vs. traditional methods)

Pooled estimates from diagnostic studies demonstrated substantial discriminative ability (pooled OR = 6.51; 95% CI 2.92–14.54). The AI subgroup demonstrated comparatively higher effect estimates (pooled OR = 9.43; 95% CI 3.38–26.33; *p* < 0.001); however, these findings were derived from a small number of studies employing heterogeneous deep learning architectures and predominantly internal validation. Accordingly, the observed effect should be interpreted as exploratory rather than indicative of definitive diagnostic superiority. Within conventional imaging modalities, CBCT showed a consistent diagnostic advantage over two-dimensional panoramic radiography (pooled OR = 3.13; 95% CI 2.34–4.94; I² = 0.0%). As illustrated in Fig. 4, diagnostic performance varied across methodological approaches; nevertheless, current evidence supports the use of AI as an adjunct to established imaging techniques rather than a replacement, pending further large-scale, externally validated investigations. Pooled diagnostic estimates shown in Fig. 4 were derived using a fixed-effects model, given the absence of substantial heterogeneity (I² = 0.0%).

Variability in diagnostic thresholds contributed to between-study heterogeneity; however, HSROC-based modeling indicated that such threshold differences did not substantially affect the overall direction of pooled diagnostic performance.

#### Predictive studies

Predictive studies yielded a pooled effect size of OR = 1.83; 95% CI 1.41–2.33, indicating a modest association between imaging or clinical indicators and outcomes. Most predictive models were internally validated only, while externally validated models were few and analysed separately in the main results. Although discrimination metrics were commonly reported, calibration performance was rarely assessed and was often incomplete. Consequently, calibration performance could not be robustly synthesised quantitatively, limiting evaluation of absolute risk estimation and clinical transportability.

**Fig. 5:**
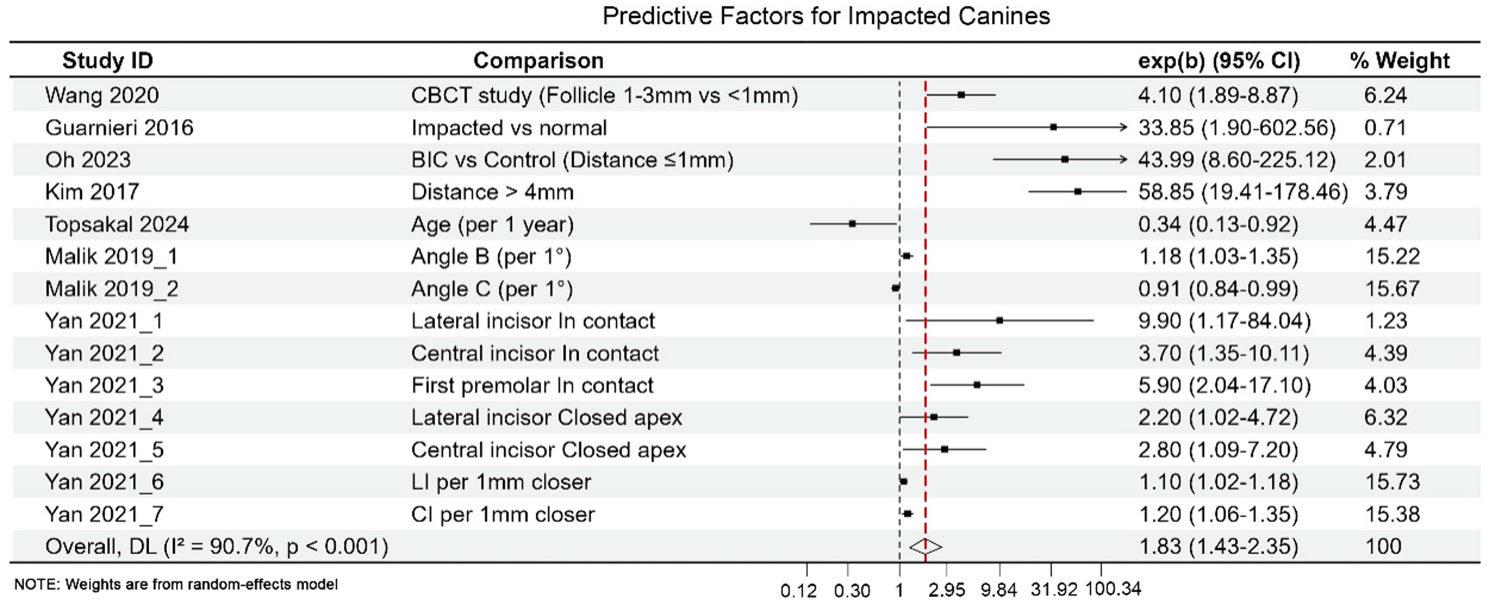
Predictive performance comparison across measurement indicators

Several predictive studies have emphasized spatial parameters central to clinical decision-making, including the degree of overlap between the impacted canine and the lateral incisor, angular relationships relative to adjacent teeth or reference planes, and the canine’s position relative to the midline. Among these, measures reflecting physical proximity and overlap consistently demonstrated stronger associations with adverse outcomes and treatment complexity, supporting their prognostic relevance across different study designs. Among these predictors, inter-root or inter-tooth contact distance demonstrated the highest predictive value (pooled OR = 4.99; 95% CI 3.06–8.97; I² = 0.0%), particularly the minimum distance between the impacted canine and the lateral incisor, which showed stable performance across studies (Fig. 5). For predictive studies (Fig. 5), a random-effects model was applied due to moderate between-study heterogeneity (I² > 50%), and pooled estimates should therefore be interpreted as average effects across heterogeneous study settings. Other geometric or angular measurements also demonstrated statistical associations, but with markedly higher heterogeneity. Evidence for AI-based predictive models remains limited, with small sample sizes and insufficient data for robust subgroup analyses. Existing findings suggest that traditional geometric and distance-based features remain advantageous due to their reproducibility and interpretability. Accordingly, predictive findings are interpreted primarily in terms of relative discrimination, with limited inference regarding calibration or real-world clinical reliability.

#### Consistency across overall and subgroup analyses

**Fig. 6:**
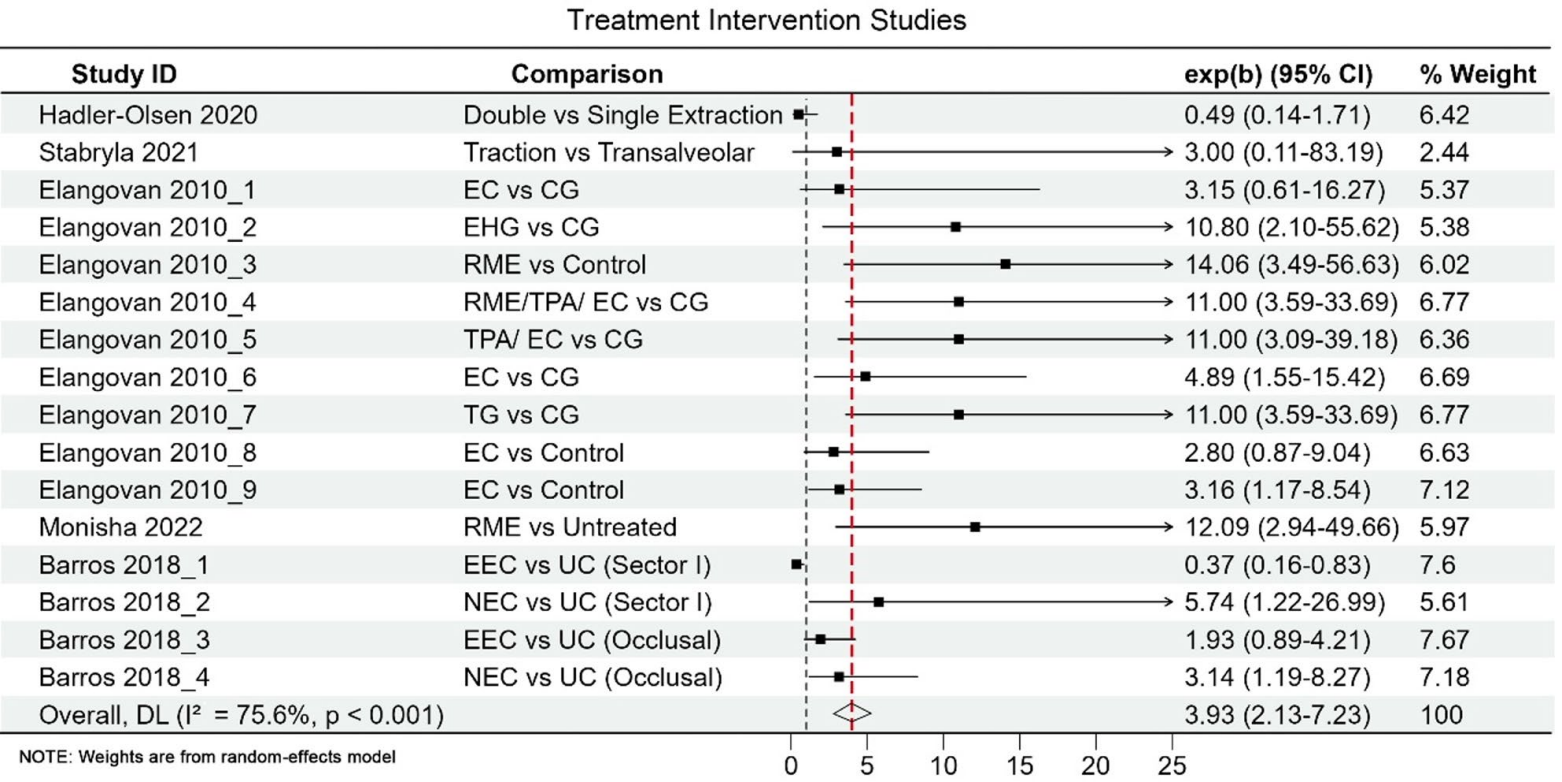
Effectiveness of therapeutic interventions

Therapeutic studies reported a pooled effect size of OR = 3.92; 95% CI 2.13–7.23, indicating that active interventions outperform conservative management strategies. Rapid maxillary expansion (RME) showed a particularly strong and consistent effect (pooled OR = 11.18; 95% CI 3.74–33.43; I² = 2.2%; *p* = 0.502), as shown in Fig. 6, suggesting that RME can markedly improve spontaneous eruption success under appropriate indications. No randomized or controlled studies have yet evaluated AI-assisted treatment planning or therapeutic decision-making. Therefore, direct comparisons between AI and traditional therapeutic approaches remain infeasible. Current evidence primarily reflects traditional intervention strategies, indicating that AI-assisted therapeutic optimization remains at an exploratory stage. Therapeutic effect estimates presented in Fig. 6 were generated using a fixed-effects model, reflecting low heterogeneity among included studies (I² = 2.2%). Although rapid maxillary expansion demonstrated a strong pooled association with spontaneous eruption, indications for expansion varied substantially across studies, and patient selection criteria were not uniform, warranting cautious interpretation of the magnitude of effect. The review by Elangovan et al. was included for contextual reference only and did not contribute primary data to the pooled therapeutic effect estimates shown in Fig. 6.

#### Consistency across overall and subgroup analyses

Subgroup analyses by study type demonstrated statistically significant differences (predictive: pooled OR = 1.83; therapeutic: pooled OR = 3.92; diagnostic: pooled OR = 6.51; AI subgroup: pooled OR = 9.43;between-group heterogeneity *p* = 0.002), consistent with the scenario-specific findings above. An overall pooled odds ratio (OR = 3.27; 95% CI 2.60–4.11; I² = 90.0%) was calculated as a descriptive summary across outcome domains; however, due to substantial conceptual and methodological heterogeneity, clinical interpretation is based on domain-specific pooled estimates rather than the overall effect. Stratification by publication year showed comparable pooled effects before and after 2020 (3.40 vs. 3.64; between-group *P* = 0.803), suggesting temporal stability. Consequently, this estimate is presented for descriptive purposes only and should not be interpreted as a unified measure of clinical effectiveness. Domain-specific pooled estimates provide the clinically meaningful evidence base and are emphasized throughout this review.

### Sensitivity analyses and exploration of heterogeneity

Sensitivity analyses demonstrated that the principal results were robust to excluding individual studies, with consistent effect directions. Funnel plots revealed no substantial systematic bias. Exploration of heterogeneity indicated that differences in study type, imaging modality, threshold definitions, and case composition contributed to inter-study variability. In diagnostic studies, threshold dependence and sample structure differences were prominent contributors, whereas in therapeutic research, heterogeneity was driven primarily by variation in intervention strategies and study design (randomized versus nonrandomized). Overall, these sources of heterogeneity did not materially alter the direction or significance of the main conclusions.

## Discussion

This systematic review and meta-analysis, synthesizing 28 independent studies, provides a comprehensive evaluation of current diagnostic, predictive, and therapeutic approaches for impacted canines and compares the performance of conventional techniques with that of AI methods. All three categories of studies demonstrated measurable effects; however, notable heterogeneity was observed across study designs, case compositions, and imaging modalities. Consequently, interpretation of the pooled estimates requires careful alignment with the clinical settings in which these methods are applied.

The high overall heterogeneity observed in this review reflects the integration of diagnostically, prognostically, and therapeutically distinct evidence streams. Such heterogeneity limits the clinical interpretability of a single pooled estimate and reinforces the importance of domain-specific synthesis. Accordingly, conclusions in this review are based on stratified analyses rather than on the overall pooled effect, which is presented for descriptive completeness only.

In the diagnostic domain, CBCT showed a consistent advantage over two-dimensional imaging in identifying spatial relationships between impacted canines and adjacent structures. This finding aligns with the known anatomical benefits of three-dimensional imaging and with the low heterogeneity observed in CBCT-based comparisons. AI-based diagnostic models showed encouraging effect estimates in selected studies; however, the current evidence remains limited by small sample sizes, retrospective designs, heterogeneous model architectures, and predominantly internal validation. Accordingly, AI findings should be interpreted as preliminary and context-specific. At present, AI is best positioned as an adjunct to clinician interpretation rather than a replacement for established imaging-based diagnosis.

From a clinical standpoint, spatial parameters remain fundamental to both diagnosis and prognosis in the management of impacted canines. The degree of overlap between the impacted canine and the lateral incisor, angular deviation from the dental arch, and proximity to the midline directly influence the risk of root resorption, treatment complexity, and eruption potential. While advanced imaging and AI-based tools may assist in quantifying these relationships, their clinical value ultimately derives from improving the assessment of these established spatial determinants. Greater emphasis on standardized reporting and integration of such parameters would enhance the interpretability and clinical utility of future diagnostic and predictive studies.

For risk prediction, traditional geometric and distance-based measurements remain the most stable and interpretable indicators. Among these, contact distance has been consistently associated with treatment success and the risk of complications across multiple studies. These metrics derive directly from anatomical relationships, thereby enhancing their reproducibility in clinical practice. In contrast, current AI predictive models are few in number, employ heterogeneous methodologies, and frequently lack essential assessments such as calibration analysis or external validation. As a result, their generalizability across different populations and institutions remains uncertain. At present, AI can serve as a supplementary reference, whereas structural imaging indicators should remain the primary basis for risk assessment.

Regarding therapeutic management, the results demonstrate that active interventions generally provide greater clinical benefit than conservative strategies. Rapid maxillary expansion, when applied under appropriate indications, substantially increases the likelihood of successful spontaneous eruption and shows strong consistency across studies. Nevertheless, considerable variation in intervention criteria, patient selection, and follow-up protocols was noted across therapeutic studies. Therefore, the pooled estimates should be interpreted as reflecting overall trends rather than precise recommendations for specific clinical decisions. Importantly, high-quality evidence evaluating the contribution of AI in treatment planning or pathway optimization remains absent. The clinical value of AI in therapeutic decision-making, therefore, cannot be determined based on the current literature.

This review indicates that conventional imaging modalities and structural indicators remain the primary and most reliable basis for clinical decision-making in the management of impacted canines. Although AI demonstrates potential in several aspects, its current role is better positioned as complementary rather than substitutive. Whether its advantages can be consistently reproduced in real-world clinical workflows requires further validation. Future research should prioritize large-scale, multicenter, prospective studies, along with improvements in model interpretability and integration with clinical workflows, to clarify the actual contributions of AI to the precision management of impacted canines.

The findings of this review should be interpreted within their respective outcome domains. Diagnostic accuracy, predictive modeling, and therapeutic effectiveness are distinct clinical constructs, and aggregating them into a single inferential estimate is not clinically meaningful. Accordingly, the overall pooled effect is retained for descriptive completeness only, while domain-specific analyses provide the basis for clinical interpretation. The use of HSROC modeling further supports the robustness of diagnostic accuracy estimates by accounting for inter-study variability in thresholds.

From a clinical implementation perspective, performance metrics alone are insufficient. CBCT provides reliable three-dimensional assessment but introduces higher cost and radiation exposure; therefore, use should remain indication-based and aligned with ALARA principles, especially in younger patients. AI tools may improve reporting consistency and workflow efficiency, but real-world implementation depends on external validation, clinician training, software interoperability, data governance, and regulatory oversight. A practical near-term model is selective deployment in complex cases with human oversight, followed by staged expansion after local validation.

Compared with previous systematic reviews, which often focused on isolated domains, this review provides a broader and methodologically stratified synthesis across diagnostic, predictive, and therapeutic evidence. It also strengthens interpretation by explicitly separating domain-level conclusions, incorporating hierarchical diagnostic modelling for threshold variability, and clarifying the current maturity limits of AI evidence. Therefore, the present review advances the field not by claiming replacement effects, but by defining where evidence is robust, where it remains exploratory, and where future research should be prioritised.

Overall, conventional imaging modalities and structural indicators remain the most reliable basis for current clinical decision-making in impacted canine management. AI shows potential, but its present role is complementary. Future work should prioritise large multicentre prospective studies, rigorous external validation, consistent calibration reporting, and integration studies that evaluate clinical impact, safety, and workflow performance in real practice.

Finally, all inferences in this review are aligned with statistical uncertainty. Where effect estimates were imprecise or heterogeneous, conclusions are framed as suggestive rather than confirmatory, with greater emphasis on consistency, plausibility, and clinical context than on point estimates alone.

### Strengths and limitations

The strengths of this systematic review and meta-analysis include strict adherence to PRISMA 2020 guidelines, use of a predefined PICOS framework, and application of domain-specific quantitative synthesis for diagnostic, predictive, and therapeutic studies. Appropriate risk-of-bias tools were employed according to study design, and diagnostic accuracy was synthesized using hierarchical models to account for threshold effects. Importantly, evidence related to AI was interpreted conservatively with explicit consideration of model heterogeneity, validation strategy, and clinical maturity.

Several limitations should be acknowledged. First, substantial methodological and clinical heterogeneity was observed across study designs, imaging modalities, outcome definitions, and analytical approaches, limiting the interpretability of pooled estimates beyond domain-specific conclusions. Second, the predominance of retrospective and single-center studies, often with short follow-up periods, increases susceptibility to selection bias and restricts long-term clinical inference. In impacted canine research, variability in impaction patterns, spatial relationships, and treatment decision pathways further limits causal interpretation in the absence of prospective stratification.

Third, the evidence base for AI-based diagnostic and predictive models remains immature, characterized by small sample sizes, heterogeneous architectures, limited external validation, and inconsistent reporting of calibration. Accordingly, AI-related findings should be regarded as exploratory and adjunctive, rather than clinically decisive. The integration of conventional clinical studies and emerging AI investigations within a single review, while necessary to reflect current research trends, limits direct comparability between these evidence streams despite the use of domain-specific analyses.

Fourth, restricting the search period to studies published since 2015 may have led to an underrepresentation of earlier therapeutic and interceptive evidence, particularly for long-established treatment approaches. This decision was made to ensure relevance to contemporary imaging and computational methods, but should be considered when interpreting therapeutic conclusions. The use of a minimum sample size threshold may also have excluded smaller exploratory studies, prioritizing stability of pooled estimates at the expense of breadth.

Finally, none of the included studies reported patient-reported outcome measures (PROMs), treatment-related harms, or long-term patient-centered outcomes, limiting assessment of real-world effectiveness. In addition, inclusion of studies with varying methodological quality and evidence of publication bias suggests that pooled effect sizes may be inflated. Therefore, greater weight should be placed on consistency and domain-specific findings rather than on overall pooled estimates alone. When imaging modalities involving ionizing radiation, such as CBCT, are considered, clinical interpretation must also align with radiation protection principles (ALARA), particularly in younger patients.

## Conclusion

This systematic review and meta-analysis, synthesizing 28 independent studies, provides a consolidated assessment of the current evidence on the diagnosis, risk prediction, and treatment management of impacted canines. Conventional imaging techniques and structural measurement-based indicators continue to demonstrate the highest stability and reliability in clinical practice and remain the primary basis for diagnostic and therapeutic decision-making. Although AI technologies have shown promising potential in selected studies, particularly in image recognition and data processing, current evidence is limited by the absence of external validation and insufficient assessment in real-world clinical settings. At this stage, AI is more suitably positioned as a complementary tool rather than a replacement for established diagnostic or predictive approaches.

Despite substantial variability in study designs, case compositions, and outcome definitions, the pooled results consistently underscore the central value of imaging-based and structural features in the management of impacted canines. Accordingly, where pooled estimates were imprecise or not statistically conclusive, we interpret findings as suggestive associations rather than definitive superiority. A key unresolved question is whether AI models can maintain stable performance across different imaging modalities, diverse clinical scenarios, and multicenter datasets. Advancing the clinical integration of AI will require large-scale, high-quality, and representative prospective studies, as well as improvements in interpretability and workflow integration. Overall, the precision management of impacted canines continues to rely on established conventional methods, whereas AI presents meaningful potential that has yet to be substantiated through rigorous validation. The findings of this review provide an initial framework for defining the role of AI within this domain and highlight the critical directions for future high-quality research.

Future research should prioritize larger, prospectively designed studies with longer follow-up, while ensuring that the use of CBCT and related imaging techniques is justified and optimized in accordance with ALARA principles. Future studies should include PROMs, adverse outcomes, and long-term clinical endpoints to better inform patient-centered decision-making.

## Supplementary Information

Supplementary Material 1.

## Data Availability

All data generated or analysed during this study are included in this article and supplementary material. Any additional information related to the datasets analysed during the current study is available from the corresponding author upon reasonable request.

## Abbreviations

AI: Artificial Intelligence
ALARA: As Low As Reasonably Achievable
AUC: Area Under the Curve
CBCT: Cone-Beam Computed Tomography
CI: Confidence Interval
CNN: Convolutional Neural Network
DCA: Decision Curve Analysis
DOR: Diagnostic Odds Ratio
FN: False Negative
FP: False Positive
HSROC: Hierarchical Summary Receiver Operating Characteristic
I²: Higgins’ Heterogeneity Statistic
MeSH: Medical Subject Headings
OPG: Orthopantomogram
OR: Odds Ratio
PICOS: Population, Intervention, Comparator, Outcomes, Study Design
PRISMA: Preferred Reporting Items for Systematic Reviews and Meta-Analyses
PROBAST: Prediction model Risk Of Bias ASsessment Tool
PROMs: Patient-Reported Outcome Measures
QUADAS-2: Quality Assessment of Diagnostic Accuracy Studies-2
RME: Rapid Maxillary Expansion
RoB 2.0: Risk of Bias 2.0
ROBINS-I: Risk Of Bias In Non-randomized Studies of Interventions
RR: Risk Ratio
HR: Hazard Ratio
TN: True Negative
TP: True Positive
