# Supplementary material for: Diagnostic, predictive, and therapeutic approaches for impacted canines: a systematic review and meta-analysis
Source: BMC Oral Health. 2026 Mar 10;26:682. doi: 10.1186/s12903-026-08072-5 (PMC13088651; doi:10.1186/s12903-026-08072-5)
Supplement: Supplementary file 1 — Supplementary Material 1. [file 12903_2026_8072_MOESM1_ESM.docx]

**Supplementary Material**

**Table S1. Full electronic search strategy**

| **Database** | **Full search string** | |
| --- | --- | --- |
| PubMed | (“impacted canine” OR “canine impaction”) AND (diagnos* OR predict* OR prognos* OR treatment OR management) AND (“cone beam computed tomography” OR CBCT OR panoramic) AND (“artificial intelligence” OR “machine learning” OR “deep learning”) |  |
| Embase | (‘impacted canine’/exp OR ‘canine impaction’) AND (‘diagnosis’/exp OR ‘prediction’/exp OR ‘treatment’/exp) AND (‘cone beam computed tomography’ OR ‘panoramic radiography’) AND (‘artificial intelligence’ OR ‘machine learning’) |  |
| Web of Science | TS=(impacted canine* AND (diagnos* OR predict* OR treatment*) AND (CBCT OR panoramic) AND (“artificial intelligence” OR “machine learning”)) |  |
| Cochrane Library | (“impacted canine” OR “canine impaction”) AND (diagnosis OR prediction OR treatment) |  |

**Supplementary Table S2.Data extraction summary of included studies (n = 28)**

| **Author (Year)** | **Domain** | **Modality / Intervention (Comparison)** | **Effect Metric (OR, 95% CI)** | **Primary Outcome** | **Risk of Bias Tool** |
| --- | --- | --- | --- | --- | --- |
| Wang (2020) | Predictive | CBCT follicle width (1–3 mm vs <1 mm) | 4.10  (1.90–8.90) | Prediction of eruption outcome | ROBINS-I |
| Guarnieri (2016) | Predictive | Impacted vs normal canine | 33.85  (1.90–602.30) | Prediction of impaction severity | ROBINS-I |
| Hadler-Olsen (2020) | Therapeutic | Double vs single extraction | 0.49  (0.14–1.70) | Treatment success | RoB 2 |
| Mousa (2023) | Diagnostic | CBCT vs OPG (root resorption) | 27.00  (2.97–247.52) | Diagnostic accuracy | RoB 2 |
| Swaity (2024) | AI-Diagnostic | AI vs semi-automated assessment | 24.00  (8.83–65.40) | Diagnostic accuracy | QUADAS-2 |
| Abdulkreem (2024) | AI-Diagnostic | Cropped vs non-cropped AI model | 3.74 (1.23–11.38) | Diagnostic accuracy | QUADAS-2 |
| Stabryla (2021) | Therapeutic | Orthodontic traction vs transalveolar | 3.00  (0.11–84.60) | Treatment success | ROBINS-I |
| Oh (2023) | Predictive | Bone–incisor contact ≤1 mm | 44.00  (8.60–225.20) | Prediction of eruption failure | ROBINS-I |
| Elangovan (2010)_1 | Therapeutic | Extraction of primary canine vs control | 3.15  (0.61–16.31) | Spontaneous eruption | RoB 2 |
| Elangovan (2010)_2 | Therapeutic | Headgear vs control | 10.80 (2.10–55.67) | Spontaneous eruption | RoB 2 |
| Elangovan (2010)_3 | Therapeutic | Rapid maxillary expansion vs control | 14.06  (3.49–56.64) | Spontaneous eruption | RoB 2 |
| Elangovan (2010)_4 | Therapeutic | RME + TPA + EC vs control | 11.00 (3.59–33.72) | Spontaneous eruption | RoB 2 |
| Elangovan (2010)_5 | Therapeutic | TPA + EC vs control | 11.00  (3.09–39.21) | Spontaneous eruption | RoB 2 |
| Elangovan (2010)_6 | Therapeutic | EC vs control | 4.89  (1.55–15.44) | Spontaneous eruption | RoB 2 |
| Elangovan (2010)_7 | Therapeutic | Transpalatal traction vs control | 11.00  (3.59–33.72) | Spontaneous eruption | RoB 2 |
| Elangovan (2010)_8 | Therapeutic | EC vs control (alternative cohort) | 2.80  (0.87–9.06) | Spontaneous eruption | RoB 2 |
| Elangovan (2010)_9 | Therapeutic | EC vs control (alternative cohort) | 3.16  (1.17–8.55) | Spontaneous eruption | RoB 2 |
| Kim (2017) | Predictive | Distance >4 mm | 58.90  (19.20–180.00) | Prediction of eruption failure | ROBINS-I |
| Alayyash (2024) | Diagnostic | CBCT vs panoramic accuracy | 3.50  (1.15–10.61) | Diagnostic accuracy | QUADAS-2 |
| Topsakal (2024) | Predictive | Age (per 1-year increase) | 0.34  (0.13–0.93) | Prediction of eruption | ROBINS-I |
| Malik (2019)_1 | Predictive | Angular parameter B (per 1°) | 1.18  (1.02–1.35) | Prediction of eruption | ROBINS-I |
| Malik (2019)_2 | Predictive | Angular parameter C (per 1°) | 0.91  (0.84–0.99) | Prediction of eruption | ROBINS-I |
| Hajeer (2022)_1 | Diagnostic | CBCT vs 2D (proximity) | 14.73  (5.72–37.99) | Diagnostic accuracy | QUADAS-2 |
| Hajeer (2022)_2 | Diagnostic | CBCT vs 2D (resorption) | 2.78  (1.61–4.79) | Diagnostic accuracy | QUADAS-2 |
| Hajeer (2022)_3 | Diagnostic | CBCT vs 2D (palatal position) | 8.00  (3.03–21.06) | Diagnostic accuracy | QUADAS-2 |
| Yan (2021)_1 | Predictive | Lateral incisor contact | 9.90  (1.23–79.73) | Prediction of impaction | ROBINS-I |
| Yan (2021)_2 | Predictive | Central incisor contact | 3.70  (1.37–10.19) | Prediction of impaction | ROBINS-I |
| Yan (2021)_3 | Predictive | First premolar contact | 5.90  (1.99–17.18) | Prediction of impaction | ROBINS-I |
| Yan (2021)_4 | Predictive | Lateral incisor closed apex | 2.20  (1.04–4.73) | Prediction of impaction | ROBINS-I |
| Yan (2021)_5 | Predictive | Central incisor closed apex | 2.80  (1.09–7.25) | Prediction of impaction | ROBINS-I |
| Yan (2021)_6 | Predictive | Lateral incisor distance (per mm) | 1.10  (1.03–1.19) | Prediction of impaction | ROBINS-I |
| Yan (2021)_7 | Predictive | Central incisor distance (per mm) | 1.20  (1.05–1.32) | Prediction of impaction | ROBINS-I |
| Monisha (2022) | Therapeutic | Rapid maxillary expansion vs untreated | 12.09  (2.92–49.99) | Spontaneous eruption | RoB 2 |
| Barros (2018)_1 | Therapeutic | Early extraction vs control (Sector I) | 0.37  (0.17–0.84) | Spontaneous eruption | ROBINS-I |
| Barros (2018)_2 | Therapeutic | No extraction vs control (Sector I) | 5.74  (1.22–27.07) | Spontaneous eruption | ROBINS-I |
| Barros (2018)_3 | Therapeutic | Early extraction vs control (occlusal) | 1.93  (0.88–4.23) | Spontaneous eruption | ROBINS-I |
| Barros (2018)_4 | Therapeutic | No extraction vs control (occlusal) | 3.14  (1.19–8.29) | Spontaneous eruption | ROBINS-I |
